# MicroRNA expression profiling of cutaneous squamous cell carcinomas and precursor lesions

**DOI:** 10.1002/ski2.360

**Published:** 2024-03-16

**Authors:** Akbor Hossain, Lisa N. Tom, Ala Melati‐Rad, Miko Yamada, Sabrina Hammerlindl, Kasturee Jagirdar, Tarl W. Prow, H. Peter Soyer, Mitchell S. Stark

**Affiliations:** ^1^ Frazer Institute The University of Queensland Dermatology Research Centre Brisbane Queensland Australia; ^2^ The Melanoma Centre South Brisbane Queensland Australia; ^3^ Skin Research Centre York Biomedical Research Institute Hull York Medical School University of York York UK; ^4^ Department of Pharmaceutical Chemistry The University of California San Francisco California USA; ^5^ Department of Dermatology Princess Alexandra Hospital Brisbane Queensland Australia

## Abstract

**Background:**

Actinic keratoses (AK) are pre‐malignant skin lesions caused by chronic sun exposure. Progression from an AK to intraepidermal carcinoma (IEC) and a cutaneous squamous cell carcinoma (SCC) is well known but the rate of transformation to an invasive SCC is highly variable. Since no definitive biomarkers are available, treatment decisions are made ad hoc.

**Objectives:**

To fully characterise our AK to SCC progression series, we performed microRNA (miRNA) microarray expression profiling of normal and photodamaged skin, as well as AKs, IEC, and invasive SCCs.

**Methods:**

The study recruited 27 patients who donated fresh biopsies of normal skin, photodamaged skin, AK, IEC, and SCC (*n* = 67 specimens). All miRbase (v.21) miRNAs were profiled to identify miRNAs related to SCC progression. miRNAs were validated using qRT‐PCR and in vitro phenotypic assays.

**Results:**

There were 234 robustly expressed miRNAs across the tissue collection, which resulted in 20 miRNA that were differentially expressed ((cor)*p* ≤ 0.05 and ≥ 10 fold) between normal skin and SCC. Hierarchical clustering all samples illustrated that AKs, IEC, and SCCs were largely indistinguishable, which confirms the premalignant status of an AK. A panel of miRNAs showed significant dysregulation between normal and photodamaged skin and AK. Importantly, we found miR‐34a‐5p and miR‐31‐5p had significant differential expression between AKs and IEC and IEC and SCC respectively. Phenotypic assays determined that the miR‐31 duplex had opposing effects on SCC cell lines which suggests that dysregulation of this duplex may be related to the dynamic control of progression of transformed keratinocytes.

**Conclusions:**

This study confirmed the continuum of AK with IEC and SCC highlighting that miRNA expression plays a role in keratinocyte transformation. Development of our putative miRNA biomarker candidates is warranted to aid in clinical management of patients experiencing high AK load to determine the most appropriate treatment.

## Graphical abstract

An actinic keratosis (AK or solar keratosis) is a precancerous skin lesion which may progress to skin cancer if left untreated. This study aimed to discover clinical biomarkers (microRNAs or miRNAs) that may identify AKs that are more prone to progress to skin cancer. This study confirmed that AKs are indeed precursors to squamous cell carcinomas (SCC), highlighting that miRNA expression plays a role in keratinocyte (skin cells) transformation. Further development of our putative miRNA biomarker candidates is warranted to aid in clinical management of patients experiencing high AK load to determine the most appropriate treatment.

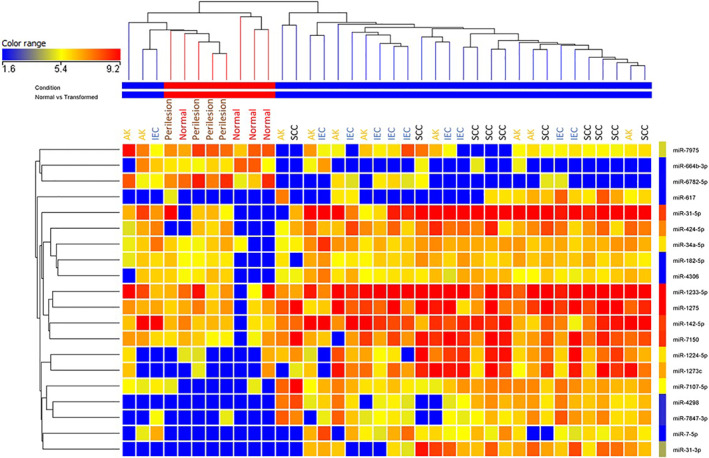

**What is already known about this topic?**

- Keratinocytic skin cancers are the most common skin cancers in Australia and other countries with high levels of UV index. Actinic keratosis (AK) is a precursor of cutaneous squamous carcinomas (SCC), but the transformation rate is highly variable. Many people experience elevated AK load due to cumulative UV exposure.

**What does this study add?**

- A panel of microRNAs showed significant dysregulation between normal and photodamaged skin and AK. Importantly, we found miR‐34a‐5p and miR‐31‐5p had significant differential expression between AK and intraepidermal carcinoma (IEC) and IEC and SCC respectively. In vitro phenotypic assays determined that miR‐31‐3p and miR‐31‐5p had opposing effects on SCC cell lines, suggesting that dysregulation of this miRNA‐duplex may be related to the dynamic control of progression of transformed keratinocytes from skin to AK/IEC/SCC.

## 1 INTRODUCTION

Cutaneous SCC is the second most common non‐melanoma skin cancer and represents a major healthcare cost due to its increasing global incidence.^1^, ^2^ It is considered that the emergence of invasive SCC is part of a clinical continuum that begins with an AK that progresses into a SCC. AKs are a precancerous, atypical neoplasms of epidermal keratinocytes characterised by scaly erythematous patches and/or hyperkeratotic papules.^3^ AKs (or solar keratosis) frequently occur in sun‐damaged sites of the scalp and face and it has been estimated that in the US alone, >40 million people have a diagnosed AK.^4^ In Australia, it has been reported that >40% of people over the age of 40 have a prior diagnosis.^5^ Although these lesions at either end of the spectrum are relatively easy to identify clinically, differentiation becomes more difficult in the intermediate areas (i.e. Intraepidermal carcinoma) of this spectrum.^6^ Studies that have attempted to investigate the natural history of AK lesions, and to ascertain both the rate of malignant transformation from AK to SCC, and regression of AK, rely on clinical diagnosis and varying methods of mapping of AK lesions. The general consensus amongst clinicians is that if an AK is left untreated, it may eventually progress to the non‐invasive form of SCC (IEC or in situ SCC) and ultimately progress to an invasive SCC.

The rate of malignant transformation of an AK is highly variable, with estimates ranging from 0.1% to 20% per year/per lesion.^7^ As such, identifying which AKs will progress into an invasive SCC is currently challenging. Instead, all AKs are treated through medical intervention, which may include the application of topical cream, cryotherapy, photodynamic therapy, as well as minimally destructive surgical procedures. This is becoming a key issue in clinical diagnostics, as the majority of patients have multiple AKs, with a recurrence rate of up to 60% within 12–24 months.^8^ Furthermore, the treatment for AKs with intrusive medical intervention is a major financial burden to the health care system, as AKs may regress or not progress. As such, a biomarker that can differentiate between AKs and SCC would be of benefit for diagnostic purposes, and possibly a source for new tailored treatment options.

Herein we performed a comprehensive miRNA profile of a collection of normal and photo‐damaged skin, AK, IEC, and SCC to identify a panel of miRNA that may offer clinical utility as predictive biomarkers. We also provide insight into the function of key miRNA and highlight the role they play in SCC progression.

## 2 MATERIALS AND METHODS

### 2.1 Patient specimen details

This study was approved by the University of Queensland Human Research Ethics Committee (Brisbane, Australia; #2013000711) and was carried out in accordance with the Declaration of Helsinki. All patients were recruited and consented at the Warner Family Medical Practice (Brisbane, Australia). All patient samples suspected of having an AK, an IEC or a cutaneous SCC were included in the study. All specimens were diagnosed by an expert pathologist and only patient samples diagnosed to be an AK, IEC, or SCC, were included for this study. A demographic summary of all patient and samples can be found in Table 1.

**TABLE 1:** Patient and specimen demographics.

Prognostic factor	Totals
Patient recruited	*n* = 27
Samples collected	*n* = 67
Sex	Male (*n* = 22; 81%)
	Female (*n* = 5; 19%)
Age at collection	Male
	55–64 = 5
	65–74 = 9
	75–84 = 6
	85–94 = 2
	Female
	65–74 = 5
Histological subtype	Normal skin (*n* = 16)
	Perilesional (*n* = 16)
	Actinic keratosis (*n* = 14)
	Intraepidermal carcinoma (*n* = 11)
	Cutaneous squamous cell carcinoma (*n* = 10)

At the time of tissue collection, the specimen was bisected, with one half for routine histopathological diagnosis and the other half for laboratory analysis. The laboratory tissue was preserved in RNAlater (Thermo Fisher Scientific Inc, USA) prior to storage at −80°C. The DNA and RNA extractions were performed using the AllPrep DNA/RNA/miRNA Universal Kit (QIAGEN, Hilden, Germany). Briefly, the tissue specimens were placed in Buffer RLT Plus (QIAGEN, Hilden, Germany) and *β*‐mercaptoethanol (Invitrogen, #21985023) and then disrupted and homogenised using a Bullet Blender (Next Advance, NY, USA). The debris was pelleted, and the supernatant was collected for co‐extraction as per manufacturer's instructions. All RNA samples were quantified using Qubit RNA HS Assay kit (Invitrogen, #Q32852) as per manufacturer's protocol.

### 2.2 MicroRNA profiling and data analysis

A total of 500 ng Total RNA from normal skin (*n* = 4), perilesional (*n* = 4), AK (*n* = 10), IEC (*n* = 10) and SCC (*n* = 10) were shipped to LC Sciences (Houston, USA) to perform the miRNA microarray profiling. The profiling was performed using a custom array platform (μParaflo® technology) containing ∼2500 miRNA probes (based on miRbase v21). The quality control, labelling (Cy‐3), hybridisation, scanning, signal background subtraction and global normalisation (LOWESS) were performed by LC Sciences.

Advanced data analyses were performed in Genespring GX (Agilent Technologies, Santa Clara, USA) using the LOWESS normalised signal intensity values. All signal intensity values < 30 were considered ‘background expression’ (personal communication with LC Sciences) and changed to 0.01 prior to log2 transformation in Genespring.

### 2.3 High‐throughput RT‐qPCR

The cDNA synthesis and pre‐amplification protocol have been previously described.^9^ Briefly, custom reverse transcriptase (RT) primer pools and assay pools were created respectively consisting of the following 5× TaqMan® RT primers and 20× TaqMan® MicroRNA Assays (Life Technologies, Carlsbad, USA): miR‐1233‐5p (assay id: 476467), miR‐1275 (002840), miR‐142‐5p (assay id: 002248), miR‐31‐3p (assay id: 002113), miR‐31‐5p (assay id: 002279), miR‐182‐5p (assay id: 002334), miR‐34a‐5p (assay id: 000426), miR‐424‐5p (assay id: 000604), miR‐664b‐3p (assay id: 479148), miR‐7107 (assay id: 480541), miR‐7150 (assay id: 480845), miR‐7‐5p (assay id: 005723), miR‐7847‐3p (assay id: 480584), miR‐7975 (assay id: 466483), miR‐6782‐5p (assay id: 466455), miR‐1273c (assay id: 478677), miR‐1224 (assay id: 479547), miR‐4298 (assay id: 465290), miR‐4306 (assay id: 462492), miR‐617 (assay id: 001591) & RNU‐6 (assay id: 001973). A total of 9 ng of Total RNA was added for the subsequent cDNA synthesis reaction, followed by pre‐amplification. The pre‐amplified product was used for miRNA expression detection via the Biomark™ HD (Fluidigm®, South San Francisco, USA) system as previously described.^9^ All reactions were performed in the 96.96 Dynamic™ Array (Fluidigm®, South San Francisco, USA). Guidelines for loading and additional reagents required for the 96.96 Dynamic™ Array were followed as per the manufacturer's instructions (PN 68000130, Rev. C.).

Expression of the 20‐miRNA panel was assayed in each sample with 4 technical replicate Taqman assays. Real‐time expression data was extracted and analysed as previously described.^9^

### 2.4 Cell culture conditions

The SCC cell lines Colo‐16, SCC‐9 and SCC‐25, were kindly gifted by Dr Fiona Simpson. All cell lines were grown in growth media supplemented with 10% Foetal bovine serum (Gibco) and penicillin (100 U/mL) & streptomycin (100 μg/ml). The Colo‐16 were grown and maintained in RPMI 1640 medium (Gibco; (#11875093). The SCC‐9 and SCC‐25 cells were grown in DEMEM/F12 (Gibco # 11320033). All cells were incubated at 37°C, in a 5% CO_2_ humidifier.

### 2.5 RNA extraction

The total RNA of transfected cells was extracted using the miRNeasy Mini Kit (QIAGEN, Hilden, Germany) as per manufacturer's protocol. Briefly, cells were harvested using 0.25% trypsin solution and centrifuged for 2 min at 1200 rcf. The supernatant was discarded, and the cells were lysed in QIAZOL solution (QIAGEN, Hilden, Germany), phase separated with chloroform, and centrifuged at 12,000 g at 4°C. Total RNA was precipitated from the upper aqueous layer with 100% ethanol prior to column purification.

### 2.6 miRNAs mimics and transient transfection

The hsa‐miR‐7150 (#YM00472973), hsa‐miR‐31‐3p (#YM00470522), hsa‐miR‐31‐5p (#YM00472582) and Negative control (#YM00479902) miRCURY LNA miRNA mimics were purchased from QIAGEN (Hilden, Germany). All cell lines were transfected with Lipofectamine RNAiMAX reagents according to the manufacturer's protocol (Invitrogen, #13778150). A day prior to transfection, all cell lines were grown to 80%–90% confluency with fresh growth media. The respective miRNA mimics were diluted to a final concentration of 5 nM in Opti‐MEM I Reduced‐Serum medium (Life Technologies, #31985070) and mixed with transfection reagents. Cells were reverse transfected with cells number appropriate for well size. All cells were incubated at 37°C in a 5% CO_2_ humidifier.

### 2.7 Cellular viability assay

The sulforhodamine B (SRB) assay was utilised to quantify cellular proliferation as described previously.^10^ Briefly, ∼2000 cells/well were seeded into a 96‐well plate and reverse‐transfected with 5 nM final concentration of each miRNA mimic and negative control (5 wells/transfection). Cells were then incubated for 72 h (Colo‐16) or 96 h (SCC‐9) prior to being fixed with methanol and washed according to standard SRB assay conditions. Colourimetric quantification of the SRB dye was performed at 564 nm wavelength by using a microtiter plate reader (Multiskan GO; Thermo Fisher). Each assay was repeated in triplicate.

### 2.8 2D colony‐forming assay

Firstly, ∼1000 cells/well of Colo‐16, SCC‐25 and SCC‐9 were seeded in a 6‐well plate for 24 h. Then, the media was removed, and cells were forward transfected as per manufacturers recommendation. All three miRNA mimics and negative control assays were performed in triplicate wells with a final concentration of 5 nM. The cells were incubated at 37°C, in a 5% CO_2_ humidifier for 10 days. To visualise the colonies, the cells were fixed and stained with 4% (vol/vol) paraformaldehyde and 0.5% (vol/vol) crystal violet mixture for 30 min. Each assay was repeated in triplicate. Each plate was scanned and colonies visually inspected and counted manually.

### 2.9 Statistical analysis

GraphPad Prism version 8.0.0 for Windows (San Diego, California USA) was used for all statistical analysis. Each miRNA was assessed for expression variance using a one‐way analysis of variance (ANOVA) or Kruskal‐Wallis test. Mann‐Whitney *U*‐test were performed for pair‐wise comparisons. Predictive ability of each miRNA was evaluated using receiver operating characteristic (ROC curve) and area under the curve (AUC) or AUROC (GraphPad Prism 8). A *p*‐value ≤0.05 was considered statistically significant.

## 3 RESULTS

### 3.1 MicroRNA expression levels are dysregulated in cutaneous SCCs and precursor lesions

To find miRNAs that may be involved in the transformation of AK into SCC, we performed global miRNA expression profiling of normal skin (*n* = 4), perilesional skin (*n* = 4), AK (*n* = 10), IEC (*n* = 10), and SCC (*n* = 10) samples (Table 1), using a microarray platform that contained ∼2500 miRNAs (miRBase v21). To identify miRNAs that had robust expression levels, the microarray signal intensities were stringently filtered (Materials and Methods) which reduced the list to a total of 234 miRNAs. Next, differentially expressed (DE) miRNA that may be related to SCC progression, were identified using a volcano‐plot analysis (*p* ≤ 0.05 and ≥10 fold; corrected for multiple testing using Benjamini‐Hochberg) was performed with the group of normal skin samples (*n* = 4) compared with SCC (*n* = 10). This revealed a specific gene list of 31 miRNAs that may be related to SCC progression. To ensure that only robustly expressed miRNAs (Materials and Methods) were present in the 31‐gene list, we next performed a Venn analysis to find overlap with the prior filtered gene list (*n* = 234) based upon robust expression, which resulted in 20 miRNA genes (Table 2). Three miRNAs (miR‐664b‐3p, miR‐6782‐5p, miR‐7975) were significantly downregulated in SCC compared to normal skin, whilst 17 (Table 2) of the miRNAs were upregulated in SCC. Interestingly, 11 of 20 miRNA have not previously been associated with SCC progression.

**TABLE 2:** The list of 20 differentially expressed (DE) miRNAs in squamous cell carcinoma (SCC) from global microRNA (miRNA) expression profiling.

miRNA	Fold change	Adjusted *p*‐value	Alteration in SCC relative to normal skin	Known/Novel
miR‐6782‐5p	58	0.006	Down	Novel
miR‐664b‐3p	52	0.002	Down	Novel
miR‐7975	13	0.035	Down	Novel
miR‐31‐5p	1367	0.007	Up	11
miR‐424‐5p	148	0.008	Up	12
miR‐31‐3p	84	0.015	Up	11
miR‐1273c	66	0.019	Up	Novel
miR‐4298	61	0.044	Up	Novel
miR‐7847‐3p	53	0.029	Up	13
miR‐7107‐5p	50	0.012	Up	Novel
miR‐1224‐5p	29	0.017	Up	Novel
miR‐4306	28	0.034	Up	Novel
miR‐7‐5p	27	0.028	Up	14
miR‐617	22	0.039	Up	Novel
miR‐182‐5p	21	0.019	Up	15
miR‐1275	20	0.006	Up	16
miR‐7150	17	0.013	Up	Novel
miR‐1233‐5p	16	0.035	Up	Novel
miR‐34a‐5p	14	0.007	Up	17
miR‐142‐5p	11	0.011	Up	18

**FIGURE 1:**
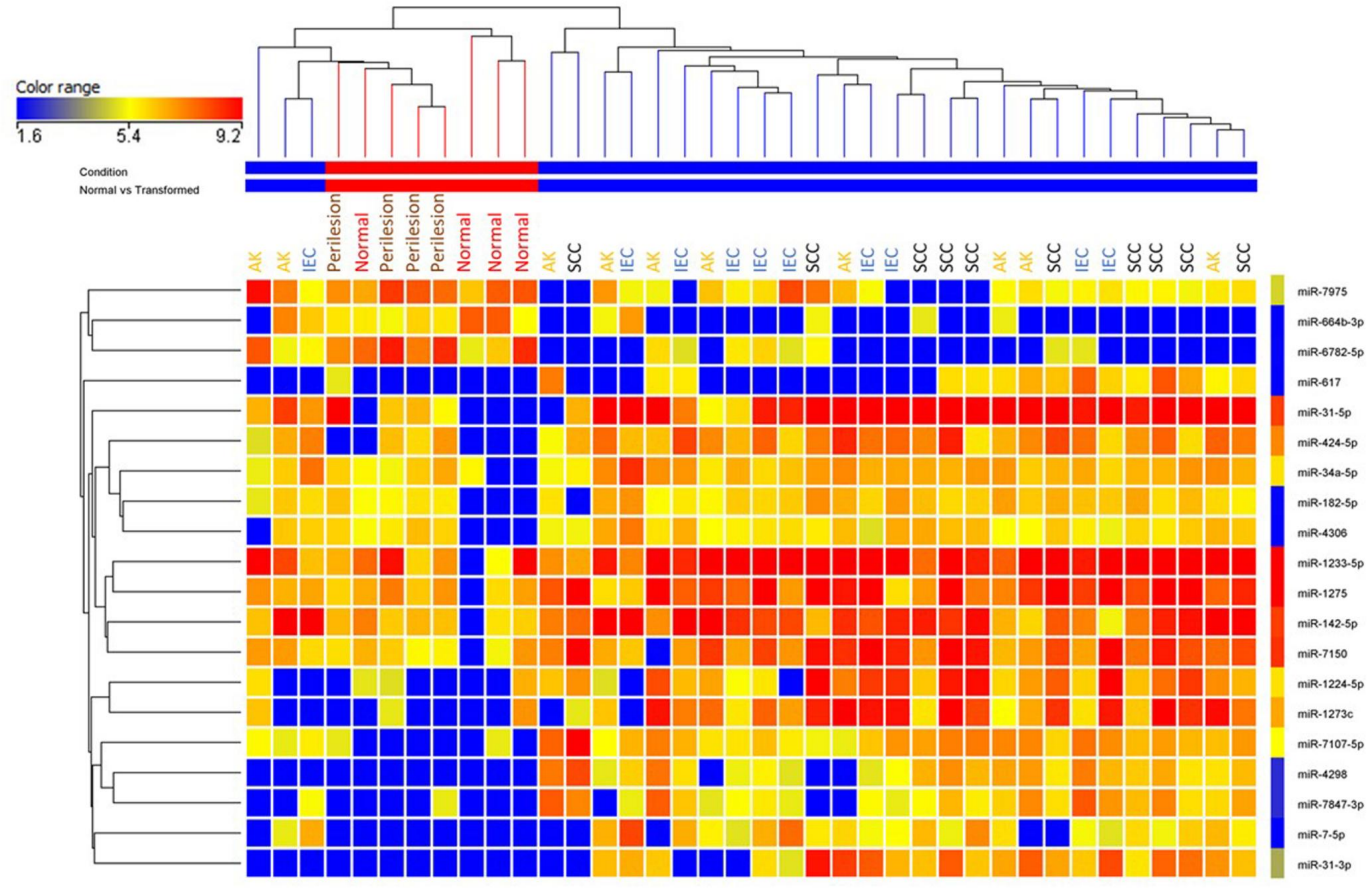
A semi supervised hierarchical clustering of 20 significantly (p(corr)≤0.05) differentially expressed (DE) microRNA (miRNA) between squamous cell carcinoma (SCC) and normal skin.

Next, we performed a semi‐supervised hierarchical clustering of the 20 DE miRNAs (Figure 1). This highlighted that there were two main clusters consisting of normal/photo‐damaged skin and lesional tissue (AK, IEC, and SCC) (Figure 1). It is important to note that the lesional tissue were not clustered according to their specific category which provide further support for the AK‐IEC‐SCC clinical continuum.

### 3.2 Quantitative real‐time PCR validation of differentially expressed miRNAs

To confirm the expression of the 20‐miRNA panel, we used a sensitive method of validation (Materials and Methods) in an extended collection (including samples present on the microarray) of skin and lesional tissue. The combined tissue cohort (*n* = 67) comprised of normal skin (*n* = 16), perilesional skin (*n* = 16), AK (*n* = 14), IEC (*n* = 11) and SCC (*n* = 10) samples. Expression was detected in all dilutions of a positive control for 14 of the 20 miRNAs (Table 3) except for miR‐617, miR‐1224, miR‐1273c, miR‐4298, miR‐4306 and miR‐6782‐5p which experienced assay failure; thus, the panel herein will be referred to as ‘SCC‐miR‐14’. The 14 expressed miRNAs along with ANOVA and multiple comparative tests are represented in Table 3 and Figure S1, with significance indicated (range, *p* = 0.0466 ‐ <0.0001). Three miRNA (miR‐7‐5p, miR‐142‐5p, and miR‐1233‐5p) showed no significance in an ANOVA across all groups, however miR‐1233‐5p did show significance in normal skin versus AK (*p* = 0.0113) (Table 3 and Figure S1).

**TABLE 3:** Differential expression of candidate miRNAs quantified in the extended validation patient samples using RT‐qPCR. Statistically significant FDR corrected *p*‐value (≤0.05) has been highlighted.

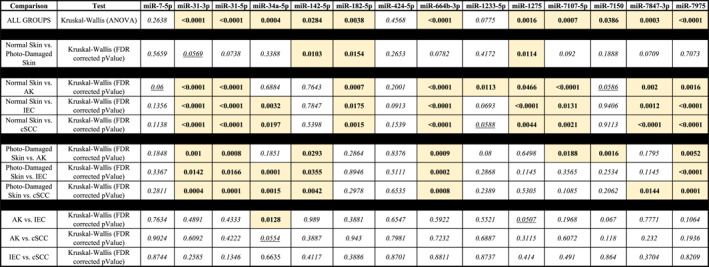

Abbreviations: AK, actinic keratosis; ANOVA, analysis of variance; FDR, False Discovery Rate; IEC, intraepidermal carcinoma; SCC, cutaneous squamous cell carcinoma.

### 3.3 miRNA expression can distinguish normal and photo‐damaged skin from AKs, IECs or squamous cell carcinoma

Among the SCC‐miR‐14, there were several miRNAs expressed in the AK, IEC, and SCC tissues, that were significantly DE compared with normal and photo‐damaged skin (Table 3 and Figure S1). However, only miR‐34a‐5p reached significance (cor. *p* = 0.0128) when the panels expression was compared between AK and IEC and there was no significant expression difference when AK and SCCs were compared or between IECs and SCCs. These lack of significant differences between AK/IEC/SCC is not surprising given the clinical scenario with AKs known to be a precursor for IEC and SCCs. The lack of significant differences can also be illustrated in Figure 1, with no clear delineation of AKs, IECs, or SCC. We next performed area under the receiver operator curve (AUROC) analysis to determine the sensitivity and specificity of the comparisons. It is apparent that the significant differences in expression noted in Table 3 also corresponded to high AUROC scores (Table 4) in most cases. This strongly indicates that all members of SCC‐miR‐14, with the exception of miR‐7‐5p, and miR‐424‐5p, contributes to AK/IEC/SCC development.

**TABLE 4:** The area under the receiver operating characteristics (AUROC) of the candidate miRNAs in patient samples. Statistically significant *p*‐value (≤0.05) and the clinically meaningful AUROC values (AUROC ≥0.7 has been highlighted.

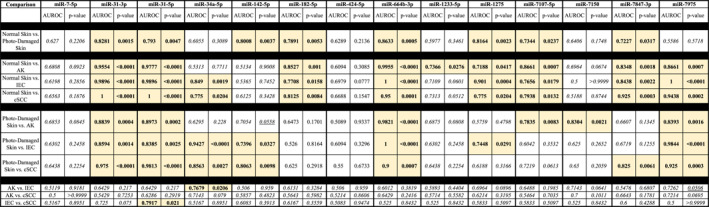

Abbreviations: AK, actinic keratosis; AUROC, area under the receiver operating characteristics; IEC, intraepidermal carcinoma; SCC, cutaneous squamous cell carcinoma.

Since, the formation of AK can lead to the IEC/SCC progression, we next explored if any of the SCC‐14 miRNAs had the potential to be used as a predictive biomarker of AK progression. Table 4 illustrates that most miRNA were involved early in the formation of AK/IEC/SCC from normal/perilesional skin, whereas only miR‐34a‐5p and miR‐31‐5p have the potential utility as predictor of AK to IEC and IEC to SCC transformation respectively.

#### 3.3.1 Overexpression of miR‐31‐3p and miR‐7150 increases cellular viability and colony formation in squamous cell carcinoma cell lines

**FIGURE 2:**
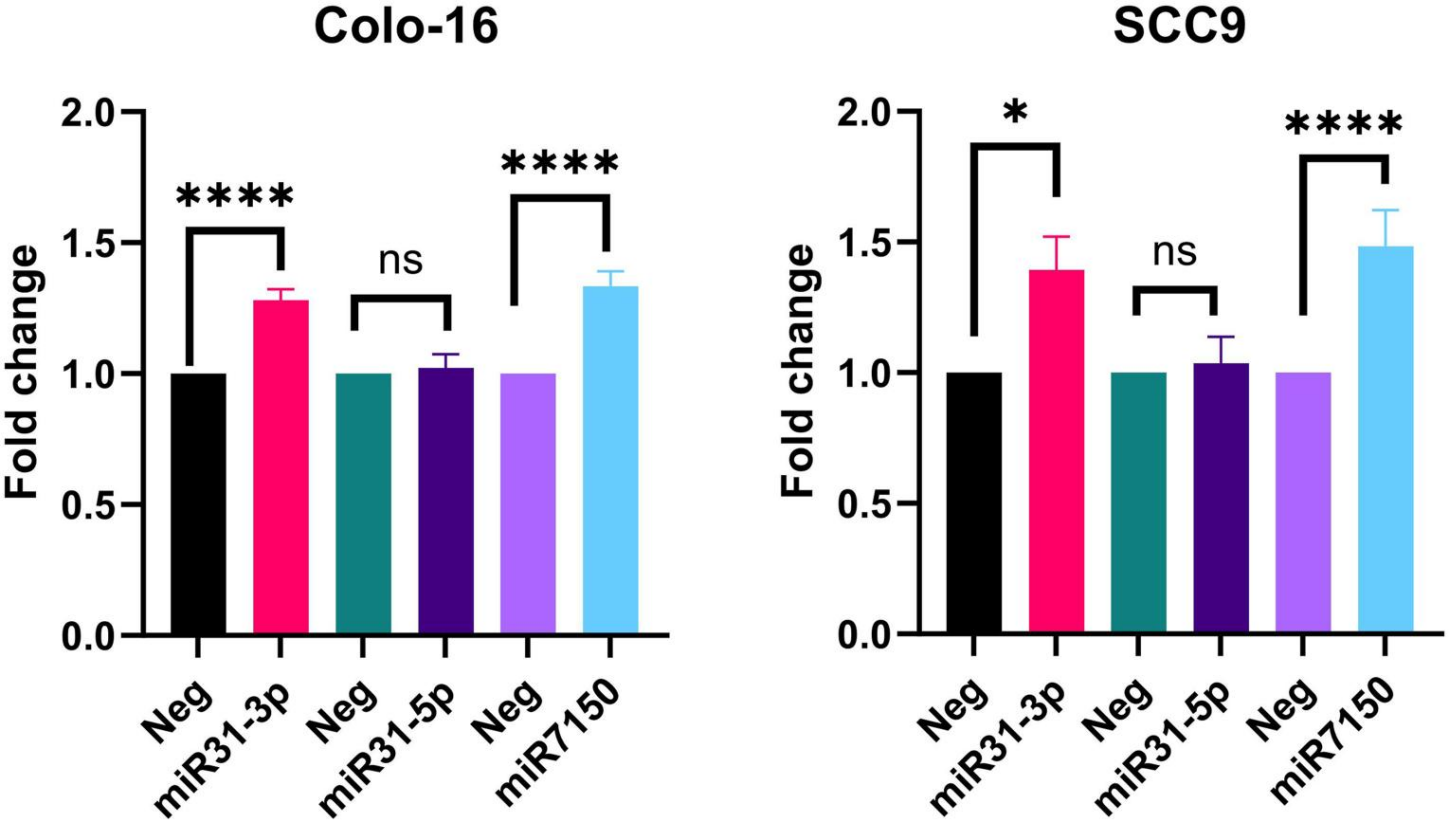
Cellular viability assay after 72 h (Colo‐16) or 96 h (SCC‐9) transfecting with 5 nM of miR‐31‐5p, miR‐31‐3p or miR‐7150 (c). Data represents three independent experiments with 5 replicates. The error bar indicates standard error of mean.

**FIGURE 3:**
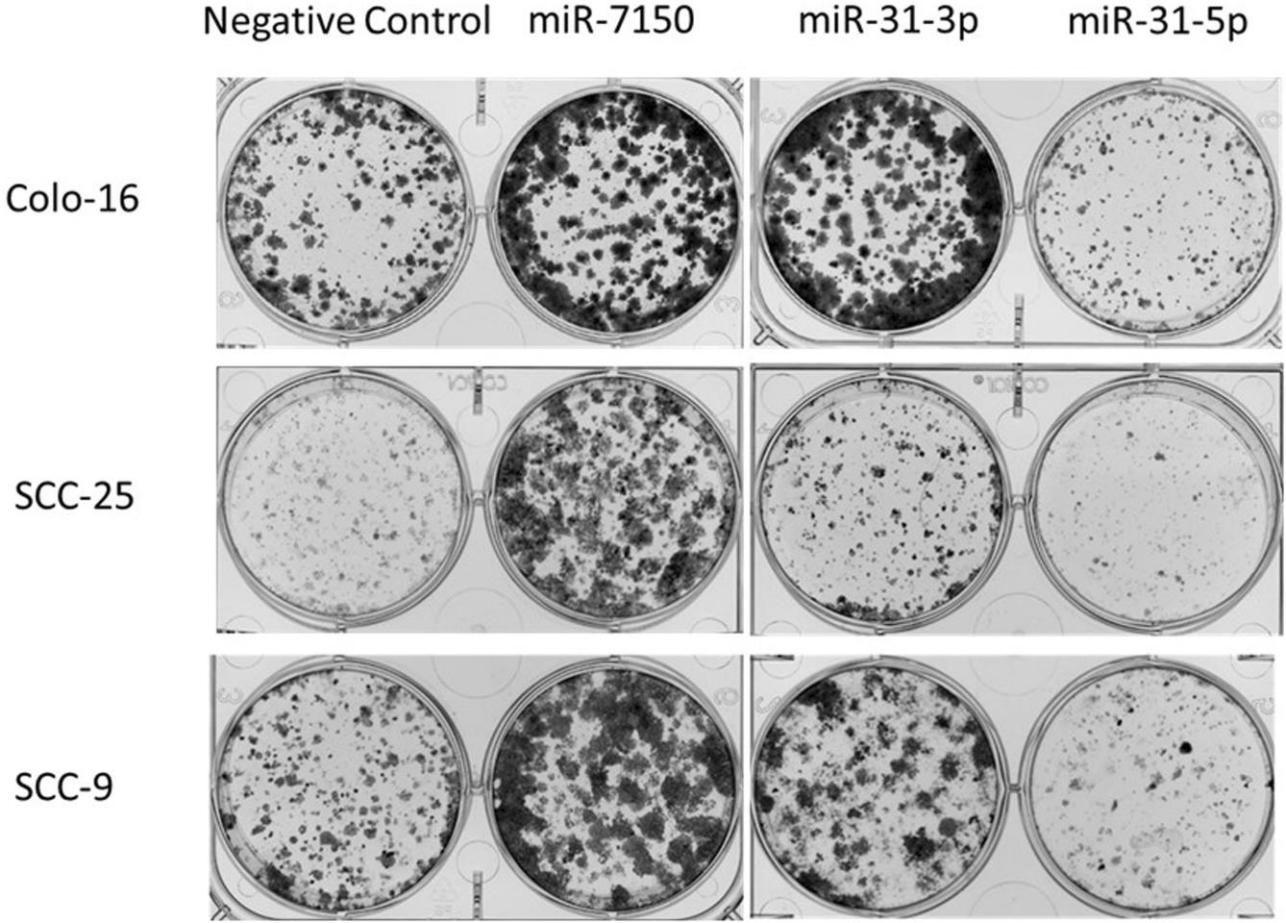
The 2D colony formation of Colo‐16, SCC‐9 and SCC‐25 cell lines transfected with 5 nM of miR‐31‐5p, miR‐31‐3p and miR‐7150 after 10 days and stained with crystal violet. The representative image is derived from triplicate experiments.

Next, we selected three miRNAs to perform functional validation as to the role they may play in SCC progression. We selected the known miRNA duplex of miR‐31 (miR‐31‐3p and miR‐31‐5p) as well as a novel miRNA (miR‐7150), as they had some of highest fold changes between normal skin and AK/IEC/SCC to perform functional assays (Table 2). Importantly, neither of these miRNAs had been specifically characterised in the setting of keratinocyte skin cancer. To assess for cellular viability, miRNA mimics and negative control were transiently transfected into two cell lines (Colo‐16 and SCC9). Interestingly, both miR‐31‐3p and miR‐7150 significantly (*p*‐value <0.05) increased cellular proliferation (Figure 2) whereas miR‐31‐5p did not show any significant changes to cellular proliferation in the assessed cell lines (Figure 2). Next, to determine if miRNA expression levels effect the ability to form 2D colonies in SCC cell lines, transfection of miR‐31‐3p and miR‐7150 led to an increase in colonies, as compared to negative control (Figure 3). In keeping with our previous observation of cell viability following overexpression of miR‐31‐5p, we observed a decrease of colony formation in all three SCC cell lines (Colo‐16, SCC9, SCC25).

## 4 DISCUSSION

It is evident that miRNAs are dysregulated in the cancer setting and their regulation of important signalling pathways play an important role in the transformation of normal cells into the cancerous state.^19^ However, there have been limited studies performed in the setting of SCC or their precursor lesions such as AK and IEC.^20^, ^21^ Moreover, we still have very limited understanding on natural history of AK formation. Here, we performed a global miRNA expression profiling in SCC and its associated precursor lesions with the goal to find potential biomarkers and the role of miRNA in SCC development.

Initially, we identified 20 miRNA that were DE between normal skin and SCC via microarray profiling. Hierarchical clustering of these miRNA highlighted that AK, IEC, and SCC were essentially indistinguishable from one another, and that they are indeed highly related. A panel of 14 (SCC‐miR‐14) were subsequently determined to have detectable expression in an extended tissue collection. Upon statistical analysis, several of the SCC‐miR‐14 panel had significant dysregulation in their expression between normal (and photodamaged) skin and SCC which highlights that normal skin and SCC have distinct miRNA expression signatures. AUROC assessment of these miRNAs revealed a high AUC score indicating that these miRNAs could easily be detected in patient samples with high specificity and sensitivity. Similarly, a panel of miRNAs showed significant dysregulation between normal skin and AK. Importantly, we found miR‐34a‐5p and miR‐31‐5p had dysregulation between AKs and IEC and IEC and SCC respectively. This has important clinical relevance, as currently no biomarkers are currently routinely available that can distinguish AKs from SCC, as such the differential expression of these miRNA may aid in histopathological diagnosis of AK highlighting their malignant potential. This corroborates previous report that AKs and SCC have distinct molecular signature based on cDNA microarray studies.^22^, ^23^

Remarkably, we found several miRNAs that show significant dysregulation between normal skin and photodamaged skin. This suggests that miRNA dysregulations occur very early on in the transformation of normal keratinocytes, leading to the formation of AK lesions and later into SCC. To further elucidate the role miRNA in SCC, we performed phenotypic assays to observe the effect of the overexpression of miR‐31‐5p, miR‐31‐3p and miR‐7150 in three SCC cell lines (Colo‐16, SCC‐9 and SCC‐25). MiR‐31 functional assays have been previously performed in the SCC context,^24^ however the individual members of the duplex (miR‐31‐5p and miR‐31‐3p) had not been studied separately. In a study by Wang *et al*,^24^ the miR‐31 precursor (pre‐miR‐31) was used which contains both strands (5′ and 3′) of the miRNA duplex. Wang *et al* ^24^ found that the overexpression of pre‐miR‐31 resulted in increased proliferation, colony formation, and cell migration and invasion. Increasingly, it has now been shown that for many miRNAs, both strands of the miRNA duplex have functional activity and in some instances, they may have an antagonist function.^25^

Overexpression of miR‐31‐3p resulted in increased cellular proliferation and increased 2D colony formation in the SCC cell lines, whereas miR‐31‐5p overexpression did not alter cell proliferation and reduced colony formation was shown. Mir‐31‐5p is known to have the higher expression, compared with miR‐31‐3p across many different tissue types, which is also observed in our study. Whilst we didn't assess the specific effect of miR‐31‐5p on invasion and migration, Wang *et al* ^24^ confirmed that the expression of pre‐miR‐31 leads to increased invasion. Based upon our observations, the opposing effects of the miR‐31 duplex suggests that their expression may be important in fine tuning the growth of transformed keratinocytes. This suggests the expression of miR‐31 may be enriched in keratinocytes and such would be suitable as a keratinocytic cancer biomarker.

Our study has a number of limitations including small sample size and lack of specimens with histopathologic evidence of SCC arising from an AK/IEC. This study has however confirmed the clinical continuum of the premalignant AK with IEC and SCC and we have highlighted that miRNA expression plays a role in keratinocyte transformation. Further development of our putative miRNA biomarker candidates is warranted to aid in clinical management of patients experiencing high AK load to determine the most appropriate treatment. This may be in the form of tape stripping^26^ or other micro‐sampling devices^27^ which can collect nucleic acids and whole cells for biomarker assessment in a non‐invasive manner.

## CONFLICT OF INTEREST STATEMENT

HPS is a shareholder of MoleMap NZ Limited and e‐derm consult GmbH and undertakes regular teledermatological reporting for both companies. HPS is a Medical Consultant for Canfield Scientific Inc., MetaOptima and Revenio Research Oy and a Medical Advisor for First Derm.

## AUTHOR CONTRIBUTIONS

**Akbor Hossain:** Data curation (equal); Formal analysis (equal); Investigation (equal); Visualisation (equal); Writing – original draft (equal); Writing – review & editing (equal). **Lisa N. Tom**: Formal analysis (equal); Investigation (supporting); Methodology (supporting); Supervision (equal); Writing – review & editing (equal). **Ala Melati‐Rad:** Methodology (supporting); Resources (lead); Writing – review & editing (equal). **Miko Yamada:** Funding acquisition (supporting); Methodology (supporting); Resources (supporting); Writing – review & editing (supporting). **Sabrina Hammerlindl:** Methodology (supporting); Writing – review & editing (supporting). **Kasturee Jagirdar:** Methodology (supporting); Resources (supporting); Writing – review & editing (supporting). **Tarl W. Prow:** Funding acquisition (supporting); Writing – review & editing (supporting). **H. Peter Soyer:** Funding acquisition (supporting); Writing – review & editing (supporting). **Mitchell S. Stark:** Conceptualisation (lead); Data curation (equal); Formal analysis (equal); Funding acquisition (lead); Investigation (lead); Methodology (lead); Project administration (lead); Supervision (lead); Visualisation (supporting); Writing – original draft (lead); Writing – review & editing (lead).

## ETHICS STATEMENT

This study was approved by the University of Queensland Human Research Ethics Committee (Brisbane, Australia; #2013000711) and was carried out in accordance with the Declaration of Helsinki.

## Supporting information

Supplementary Material

Figure S1

## Data Availability

Raw microarray data is available upon request.
