# Supplementary material for: MicroRNA expression profiling of cutaneous squamous cell carcinomas and precursor lesions
Source: Skin Health Dis. 2024 Mar 16;4(3):e360. doi: 10.1002/ski2.360 (PMC11150735; doi:10.1002/ski2.360)
Supplement: Supplementary file 1 — Supplementary Material [file SKI2-4-e360-s001.docx]

*Figure S1*

*The Box and whisker (minimum to maximum) plots represents the median normalized CT expression values* of the 14 microRNAs detected in normal skin (NS), photodamaged skin (PS), actinic keratoses (AK), intra-epidermal carcinoma (IEC), and cutaneous squamous cell carcinoma (cSCC). *= CT value normalised to the small nucleolar RNA RNU6 (10).*
