# Supplementary figures and images for: MicroRNA expression profiling of cutaneous squamous cell carcinomas and precursor lesions
Source: Skin Health Dis. 2024 Mar 16;4(3):e360. doi: 10.1002/ski2.360 (PMC11150735; doi:10.1002/ski2.360)

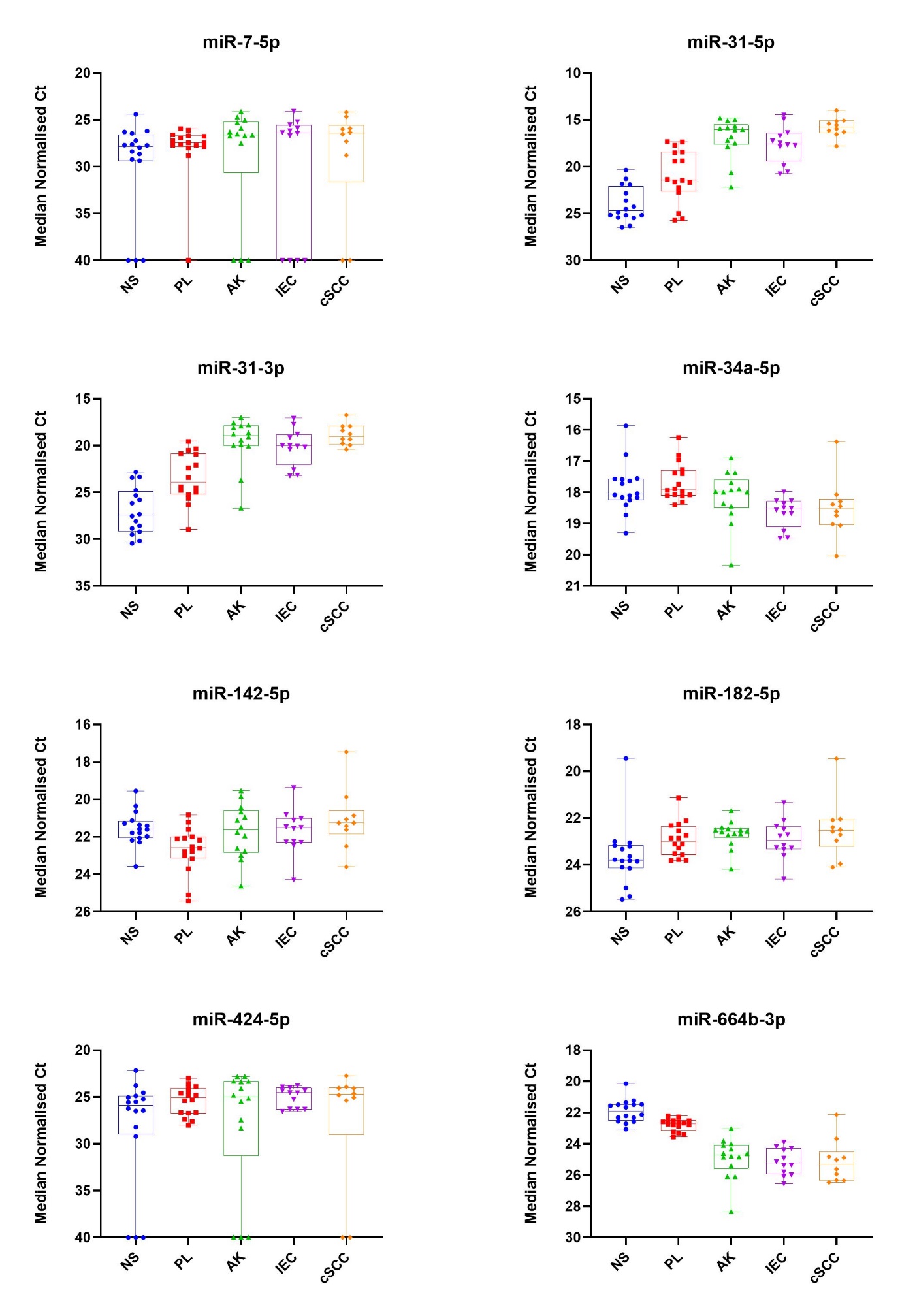


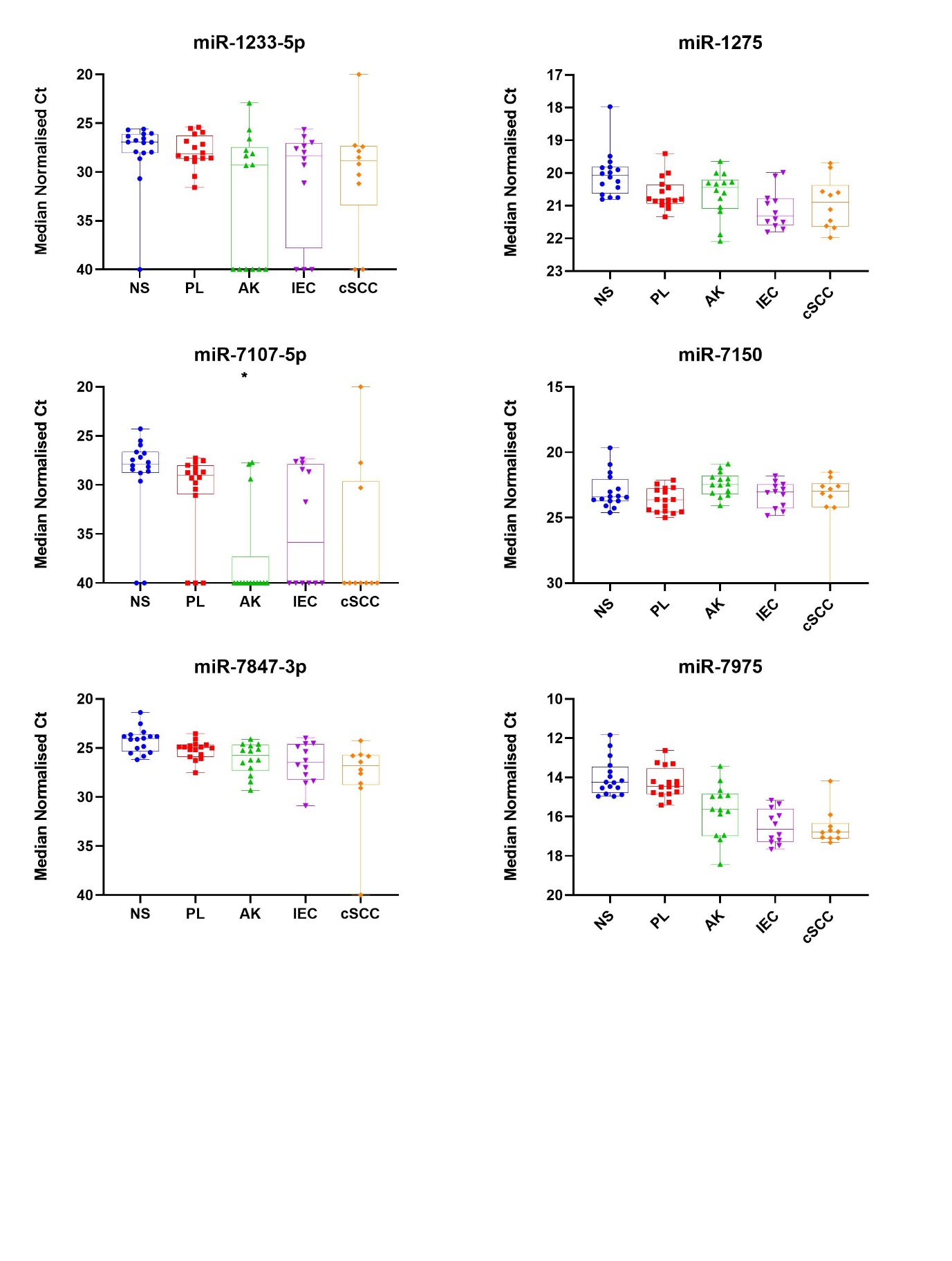

Supplement: Supplementary file 2 — Figure S1 [file SKI2-4-e360-s002.docx]
